# Fisher’s Linear Discriminant Function Analysis and its Potential Utility as a Tool for the Assessment of Health-and-Wellness Programs in Indigenous Communities

**DOI:** 10.3390/ijerph17217894

**Published:** 2020-10-28

**Authors:** Eric N. Liberda, Aleksandra M. Zuk, Ian D. Martin, Leonard J. S. Tsuji

**Affiliations:** 1 School of Occupational and Public Health, Ryerson University, Toronto, ON M5B 2K3, Canada; 2 Department of Physical and Environmental Sciences, University of Toronto, Toronto, ON M1C 1A4, Canada; aleksandra.zuk@utoronto.ca (A.M.Z.); ianmartin@mac.com (I.D.M.); leonard.tsuji@utoronto.ca (L.J.S.T.); 3 School of Nursing, Faculty of Health Sciences, Queen’s University, Kingston, ON K7L 3N6, Canada

**Keywords:** fisher’s linear discriminant function analysis, type 2 diabetes mellitus, indigenous peoples, health-and-wellness program evaluation

## Abstract

Diabetes mellitus is a growing public health problem affecting persons in both developed and developing nations. The prevalence of type 2 diabetes mellitus (T2DM) is reported to be several times higher among Indigenous populations compared to their non-Indigenous counterparts. Discriminant function analysis (DFA) is a potential tool that can be used to quantitatively evaluate the effectiveness of Indigenous health-and-wellness programs (e.g., on-the-land programs, T2DM interventions), by creating a type of pre-and-post-program scoring system. As the communities of the Eeyou Istchee territory, subarctic Quebec, Canada, have varying degrees of isolation, we derived a DFA tool for point-of-contact evaluations to aid in monitoring and assessment of health-and-wellness programs in rural and remote locations. We developed several DFA models to discriminate between those with and without T2DM status using age, fasting blood glucose, body mass index, waist girth, systolic and diastolic blood pressure, high-density lipoprotein, triglycerides, and total cholesterol in participants from the Eeyou Istchee. The models showed a ~97% specificity (i.e., true positives for non-T2DM) in classification. This study highlights how varying risk factor models can be used to discriminate those without T2DM with high specificity among James Bay Cree communities in Canada.

## 1. Introduction

Diabetes is an endocrine disorder with a worldwide distribution, occurring in developed and developing countries alike [1]. Four general categories of diabetes have been described: 1. Type 1 diabetes mellitus (T1DM) is typically caused by an autoimmune response leading to the destruction of β-cells in the pancreas, and an absolute deficiency in insulin. 2. Type 2 diabetes mellitus (T2DM) occurs from the progressive loss of insulin secretion from the β-cells in the pancreas and/or increasing insulin resistance. 3. Gestational diabetes mellitus (GDM) can appear during pregnancy when insulin insensitivity is increasing. 4. In addition, other types of diabetes also exist (e.g., drug- or chemical-induced diabetes) [2]. Approximately 346 million people are afflicted with diabetes globally, and approximately 90% of all cases are T2DM [1]. The global prevalence of T2DM has been increasing due to a number of factors (e.g., an aging population and lifestyle changes; Chen et al. [3]). 

In general, the age-adjusted prevalence of T2DM has been reported globally to be several times higher in Indigenous populations compared to their non-Indigenous counterparts [4,5]. In Canada, Indigenous (i.e., First Nations, Metis, and Inuit) peoples have disproportionately high rates of T2DM, with on-reserve First Nations people having the highest age-adjusted prevalence (17.2%) being more than three times that of the Canadian non-Indigenous population (5.0%; Government of Canada, [6]; Institute of Health Economics, [7]). In addition, the prevalence of T2DM is greater in females than males in Canadian Indigenous populations, which is opposite to the trend reported for the non-Indigenous Canadian population [6,8]. Indeed, Harris et al. [9] report age-standardized T2DM prevalence rates >20% for First Nations women and ~16% for First Nations men. Further, Canadian Indigenous persons are more likely to develop complications associated with T2DM, be hospitalized more often with diabetes-related conditions, and to die from these complications [6,7,8,9]. Thus, it is not surprising that many health promotion and prevention programs have been initiated to address the T2DM issue in Canadian Indigenous communities [7,8]. However, “there is currently a paucity of evidence on the effectiveness of [health-and-wellness programs including] interventions to prevent or treat diabetes” in Indigenous communities [10]. 

Fisher’s linear discriminant function analysis (DFA) is one potential tool that could help in the assessment of the effectiveness of health-and-wellness programs (including T2DM interventions). Discriminant function analysis is a multivariate statistical technique that uses observed predictor variables to discriminate between two or more groups identified *a priori*, and classify new observations into previously identified groups [11,12]. The number of discriminant functions is the number of *a priori* identified groups, minus one; thus, for two groups, there would be one discriminant function [13]. For the two-group case, the form of the discriminant function equation (DFE) is:

DFE = V_1_X_1_ + V_2_X_2_ + V_3_X_3_ = …V_i_X_i_ + c (1)

where DFE is the discriminant function equation, V represents the weight of the variable (i.e., the Canonical discriminant function coefficient, unstandardized), X is the discriminating variable value for the participant, i equals the number of predictor variables, and c is the constant [13]. 

Discriminant analyses have a long history of use in diabetes research (e.g., Lakowski et al. [14]; Amoah et al. [15]; Rondinelli et al. [16]). For example, Kordonouri et al. [17] used stepwise discriminant analysis to demonstrate in T1DM pediatric patients that poor glycemic control and abnormal lipid profiles were the most important variables associated with the development of retinopathy; while, elevated blood pressure was related to incipient nephropathy. More recently, in T2DM studies of archived data for Pima-Indian women living near Phoenix, Arizona, USA—discriminant analyses (generalized discriminant analysis, Polat et al. [18]; linear discriminant analysis, Dogantekin et al. [19]) were used in the first steps of two-step processes—to identify important discriminatory variables between T2DM individuals and non-T2DM women [18,19]. These variables were then entered into machine-learning programs (Least Square Support Vector Machine, Polat et al. [18]; Adaptive Network Based Fuzzy Inference System, Dogantekin et al. [19])) to classify T2DM and non-T2DM individuals in the Pima-Indian dataset. Classification accuracy (accuracy = (true positive for T2DM + true negative for non-T2DM)/(true positive for T2DM + true negative for non-T2DM + false positive for non-T2DM + false negative for T2DM); Nai-arun and Sittidech, [20]) was 82% for Least Square Support Vector Machine [18] and 85% for Adaptive Network Based Fuzzy Inference System [19]. Discriminant analysis has also been used to discriminate between non-GDM and GDM patients to identify the most important risk factors for developing GDM [21,22,23]. Although discriminant analyses have been used to identify risk factors related to the development of diabetes or its complications, to our knowledge, the discriminant analysis approach has not been suggested as an approach in assessing the effectiveness of health-and-wellness programs. In the present study, we will examine whether DFA has the potential to be used as a tool to evaluate the effectiveness of health-and-wellness programs (including T2DM interventions) in Indigenous people, by creating a type of pre-and-post-program scoring system. It should be emphasized that the tool that we present is just one component of how we might evaluate the effectiveness of on-the-land, Indigenous health-and-wellness programs. Other evaluative components could include photovoice, videovoice, and semi-directed interviews to ensure an Indigenous perspective of the effectiveness of a program. The two approaches would be complementary to each other and give a more complete understanding of the impacts of health-and-wellness programs [24]. 

## 2. Methods

### 2.1. Data Sources and Study Population

**Figure 1:**
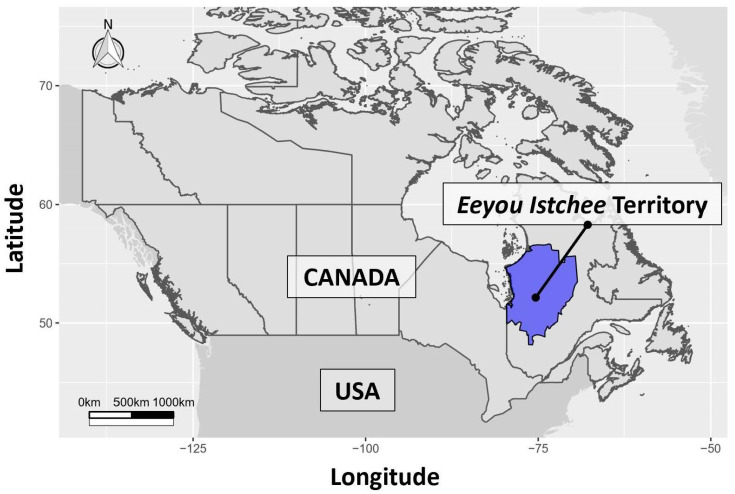
Map of the Eeyou Istchee Territory, Quebec, Canada.

The *Nituuchischaayihtitaau Aschii* multi-community environment-and-health study in the *Eeyou Istchee* Territory (Figure 1) was a multi-year study with 1730 participants from all nine First Nation communities. The *Eeyou Istchee* Territory is located in the eastern James Bay region of subarctic Quebec, Canada. This region has been classified as remote and very remote using several remoteness indices [25]. This study collected various demographic data and clinical measures including medical chart reviewed disease status of T2DM. Participants provided written informed consent in Cree, English, or French languages with ethics approval being granted by McGill University, Laval University, McMaster University, and the Cree Board of Health and Social Services of James Bay (#01013/04; #2005-067 A-1; #99.05.01; #A06-B23-05A). Beginning with all 1730 recruited participants, we excluded those who did not have all data for the following variables: age, fasting blood glucose, body mass index (BMI), waist girth, systolic blood pressure, diastolic blood pressure, HDL cholesterol, triglycerides, and total cholesterol. Further, only female and male participants over 20 years of age were included in the study. Using medical chart review, the two individuals diagnosed with T1DM were excluded from the study. Participants were then put into one of two categories: diagnosed T2DM or non-T2DM. The diagnosed T2DM category was not divided by treatment regime. However, it was noted that, of the people diagnosed with T2DM, 81% were prescribed oral hypoglycemics; 13% were prescribed insulin; and for 6%, no medication was recorded. This resulted in 755 participants, with 440 being female and 315 being male representing seven of nine Cree communities. 

### 2.2. Discriminant Function Analysis Variables

The sex, age (years), waist girth (cm), and BMI (kg/m^2^) were all assessed by an experienced clinical field nurse. Diastolic and systolic blood pressure measurements (mm Hg) were taken three times and the mean blood pressure was calculated using the last two recordings. Additionally, fasting blood samples were drawn for various lipid measures, namely HDL cholesterol (mmol/L), triglycerides (mmol/L), and total cholesterol (mmol/L) using a Vitro 950 Chemistry Station (Ortho-Clinical Diagnostics, Raritan, NJ, USA) as per Liberda et al. [26]. Fasting blood glucose (mmol/L) was also assessed using the Vitros 950 (Vitros Chemistry, Ortho-Clinical Diagnostics, Rochester, NY, USA) spectrophotometric assay system as per the manufacturer.

### 2.3. Statistical Analysis

We selected nine variables for analysis in our study: age, fasting blood glucose, body mass index (BMI), waist girth, systolic blood pressure, diastolic blood pressure, HDL cholesterol, triglycerides, and total cholesterol. These variables were identified based on the knowledge that obesity, abnormal lipid profiles, and elevated blood pressure have been associated with diabetes, and known risk factors for coronary heart disease for non-Indigenous populations [17,27,28,29,30]. Blood sample biochemistry concentration data (fasting glucose, triglycerides, HDL cholesterol, total cholesterol) were transformed as log_10_ (concentration +1) to reduce skewness and outlier leverage in analyses. Blood pressure measurements and BMI were log transformed. Participant age was not transformed. Additionally, we included log transformed waist girth in one analysis, replacing BMI as a variable. Analyses were carried out separately for females and for males as preliminary analyses showed differential variability and reliability of some variables in separating T2DM and non-T2DM, dependent upon sex. Further, it is known that the prevalence of T2DM disproportionately burdens First Nations females compared to males [9,31]. 

Initially, only age and fasting glucose were used as predictor variables in the simplest model to optimally distinguish T2DM and non-T2DM groups through discriminant functions analysis (DFA). Using these two variables, we constructed a non-stepwise Fisher’s linear DFA to separate the groups. The DFA yields new values, or DFA scores, for a synthetic, single-dimension variable for each individual, and the gradient of this variable maximizes the difference between groups. The DFA score is a linear combination of the included variables (age, and glucose, in this initial model). Probability density functions in the DFA model, based upon *a priori* group size, were used to classify all observations as either T2DM or non-T2DM. This DFA classification was then evaluated as either correct or incorrect based upon the initial clinical diagnosis. Additionally, we examined the equality of group (T2DM; non-T2DM) means of included variables in each model, with Wilk’s Lamda and its associated F-test and probability and recorded the linear discriminant function coefficients (unstandardized) for the DFA, which are the weightings for each predictor variable in the derived, single-dimension discriminant function (i.e., the DFE). 

To build successive DFA models with greater information content to potentially better discriminate between the T2DM groups—we added predictor variables that could be collected in relatively non-invasive procedures—and other variables identified by researchers as being important in their diabetes-based discriminant analysis studies (e.g., Kordonouri et al. [17]). Building on Model 1 (age and glucose), the second model added BMI, while the third model replaced BMI with waist girth. The fourth model included age, glucose, BMI, diastolic blood pressure, and systolic blood pressure, while the fifth model added total cholesterol, triglycerides, and HDL cholesterol. As a link between T2DM and cardiovascular diseases has often been reported, we also included the American Heart Association’s (AHA) risk factor suite for Metabolic Syndrome (MetS) [30] in our study for Models 6 (glucose, waist girth, systolic blood pressure, diastolic blood pressure, HDL cholesterol and triglycerides) and 7 (substituting BMI for waist girth in Model 6). With each DFA model, we again examined equality of group means and linear discriminant function coefficients to compare successive DFA models, and judged the relative discriminatory power of the various models using the classification table of subjects. Sensitivity was defined as the correct classification of participants with T2DM (i.e., sensitivity = true positive T2DM/(true positive T2DM + false negative T2DM); Polat et al. [18]; Smits, [32]). Specificity was defined as the correct classification of non-T2DM participants (i.e., specificity = true negative non-T2DM/(true negative non-T2DM + false positive non-T2DM); Polat et al. [18]; Smits, [32]). Statistical analyses were carried out using SPSS software, version 23 (IBM SPSS, Chicago, IL, USA). Figure 1 was generated using R, version 3.5.3 (R Core Team, Vienna, Austria).

## 3. Results

Table 1 summarizes age, blood biochemistry, and morphometric measures used in the analyses for female and male participants with (i.e., T2DM) and without a diagnosis of T2DM (i.e., non-T2DM). A total of 440 female Cree participated (111 T2DM; 329 non-T2DM); while, 315 males participated (52 T2DM; 263 non-T2DM). For both sexes, the youngest diagnosed T2DM cases were age 25. 

Tests of equality of group (T2DM versus non-T2DM) means were generated in the DFA and are shown for each model in Table 2. For both males and females, differences between the groups were highly significant for age (when included in the model), fasting glucose, BMI (when included), waist girth (when included), and systolic blood pressure (when included). Diastolic blood pressure was not a significant distinguishing variable for T2DM in either sex. HDL-cholesterol and triglyceride concentrations significantly distinguished between T2DM and non-T2DM groups of females, but not in the case of males. Total cholesterol concentration was indistinguishable between female T2DM and non-T2DM groups, but highly significant in separating male T2DM and non-T2DM groups. 

**Figure 2:**
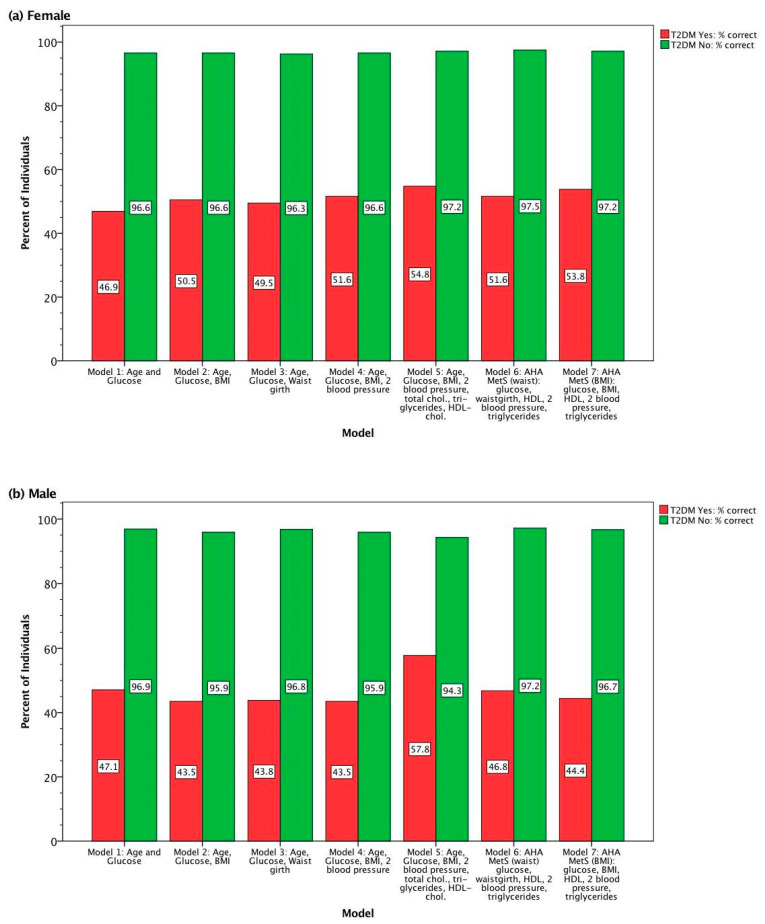
Percent correct classification of type 2 diabetes mellitus (T2DM) and non-T2DM individuals from discriminant functions analysis, for (**a**) females, and (**b**) males.

The unstandardized coefficients shown in Table 3 are the weightings of the measurement variables in each model. The linear combination of weighted variables, plus the constant shown, optimally separate T2DM and non-T2DM groups for each iterative model and are part of the DFA. The Wilk’s Lambda test of function evaluates the discriminatory effectiveness of the models. All seven models were highly significant. We examined the percent correct classification of both T2DM groups as more variables were added to successive models. The percent of participants with correctly classed T2DM and non-T2DM condition is shown in Figure 2, for females (Figure 2a) and for males (Figure 2b). Classification using each model resulted in four groups: (1). Diagnosed with T2DM, correctly classified as T2DM (i.e., true positives, Figure 2). (2). Diagnosed with T2DM, misclassified as non-T2DM (i.e., false negatives, not shown). (3). Not diagnosed with T2DM, correctly classified as non-T2DM (i.e., true negatives, Figure 2). (4). Not diagnosed with T2DM but misclassified as T2DM by the DFA model (i.e., false positives, not shown). 

In general, we found improvement in the classification accuracy of T2DM status in DFA models as more information (variables) was added (Figure 2a,b). For example, in Model 5 the variables HDL cholesterol, total cholesterol, and triglycerides were added to the variables in Model 4, the next simplest model. The addition of these variables increased the sensitivity in DFA Model 5. For most models specificity was ~97%, but minor variations in the values of this statistic may be important, as the corollary statistic is the percent of incorrect classification of non-T2DM participants. Model 6 incorporates American Heart Association (AHA; Grundy et al. [30]) MetS risk factors, and Model 7 uses the same AHA MetS risk factors but substitutes BMI for waist girth. In males, these models appear to yield similar discriminatory results compared to the simplest DFA Model 1 (age and glucose; Figure 2b); however, in females (Figure 2a), Model 6 (AHA MetS risk factors) and Model 7 (AHA MetS risk factors but substituting BMI for waist girth) suites of variables improves the correct classification of both T2DM and non-T2DM groups over Model 1. 

Table 4 presents the standardized canonical discriminant function coefficients. The standardized coefficients show all variables in the model on the same scale and allows comparison of the relative importance of each putative T2DM risk factor in determining the optimal separation of those with or without a diagnosis of T2DM. 

**Figure 3:**
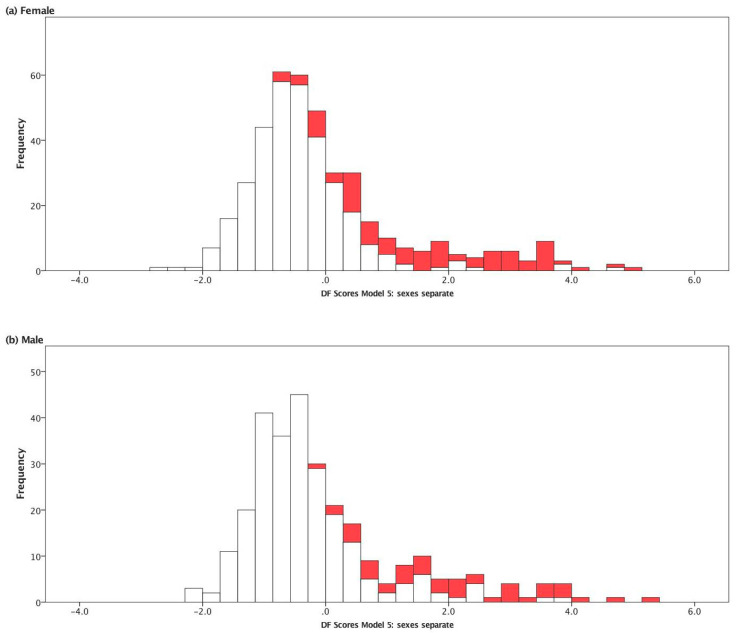
Histogram of scores from Model 5 discriminant functions analysis, for (**a**) females and (**b**) males. Solid bars: Type 2 diabetes mellitus (T2DM) participants; open bars: Non-T2DM participants.

For females, in all models, DFA scores of T2DM individuals was significantly greater (more positive values) than the DFA scores of non-T2DM individuals Figure 3). Fasting blood glucose concentration exerted the strongest positive influence in the calculation of DFA scores in each DFA model, with standardized coefficients ranging from 0.959 in the simplest (Model 1) to 0.819 in Model 5. Both waist girth and BMI had modest positive coefficients in models when they were used. Age and systolic blood pressure were minor positive forces in calculating discriminant function scores, whereas diastolic blood pressure increase would result in a decreased DFA score, as this variable had negative coefficients in each model where it was incorporated. Triglycerides were moderately strong, positive influences on DFA score, and total cholesterol concentration was a negative coefficient. The coefficients for HDL cholesterol in females were only slightly positive, indicating a small risk effect for T2DM.

Standardized coefficients for DF analysis of males shows similarities to the results for females, but with some important distinctions. Age is a stronger determinant (positive coefficient) of DF score and T2DM risk for males than was the case for females, but waist girth and BMI showed lower positive coefficient values in the case of males (Table 4). The influence of systolic blood pressure on DFA scores in males, while still positive, was comparable to that of females only in the MetS-based models (Models 6 and 7), and the negative values for diastolic blood pressure influence also were only of note in the AHA models where total cholesterol was not included. HDL cholesterol was found to be a slightly positive driver of DFA scores in males (Models 6 and 7), and more so in Model 5. Total cholesterol was the most important negative influence on DFA scores in males where used (Model 5). Triglyceride concentrations were a moderate positive influence in Model 5, only in conjunction with the inclusion of total cholesterol in the model; MetS-suite models (6 and 7) reversed the influence of triglycerides in the absence of total cholesterol measurement. This phenomenon was not observed for female DFA models. 

The histograms of the distribution of Model 5 DFA scores are shown in Figure 3 for each sex. A similar figure for Model 2 is included in Appendix A. Model 5, which incorporated age, glucose, BMI, systolic and diastolic blood pressure, and a suite of cholesterol-related measures (total cholesterol, HDL cholesterol, triglycerides), was the best suite of clinic-measurable variables for classifying most individuals (i.e., classification accuracy).

## 4. Discussion

In the present study, DFA models were assessed for their classification accuracy, sensitivity, and specificity. Classification accuracy was in the range of 70% (male, Model 2) to 76% (male, Model 5; Figure 2), but more importantly, the specificity was ~97% for the models (Figure 2). This means that the models rarely misclassified non-T2DM individuals. It is assumed that the ~3% of misclassified people would be at greater risk for developing T2DM (or may have undiagnosed T2DM), because the models classified them as T2DM based on the suite of characters of each model. The sensitivity of the models (44–58%) were much lower than the specificity of the models (97%), as was expected. We assume that because patients diagnosed with T2DM that are under clinical care (and compliant with their treatment regimen) can make improvements in the health parameters measured and used in the models, to the extent that some patients would appear to be non-T2DM in the models. Thus, the percentages of misclassified T2DM participants in the models may demonstrate a measurable positive response to treatment regimes and adherence of patients to these regimes, and/or modification of behavior (e.g., lifestyle interventions). Important for the objective of the present study, a DFA score for any of these models presented can be calculated for other Cree individuals in James Bay who were not part of the initial analysis. This can be accomplished by taking their measured values for the same variables used in a particular model and substituting these into the DFA (using the appropriate canonical discriminant function unstandardized coefficient, Table 3) for either females or males, as appropriate. These *a posteriori* DFA scored individuals can then be placed on a continuum from non-T2DM to T2DM (Figure 3 and Appendix A), and can be assessed for program effectiveness, when a baseline DFA score is compared to post-program DFA score for the exact same individual. In short, the effectiveness of a health-and-wellness program can be monitored by repeating this projection exercise with the same individual over time. 

In Canada, health-and-wellness programs aimed at decreasing the incidence of T2DM in Indigenous communities—and limiting the complications associated with the progression of T2DM—can take on many forms [6,8]. For example, typical interventions include walking clubs, weight-loss groups, and community gardens; while others incorporate traditional Indigenous food harvesting activities, canoeing, and dancing [6]. These types of community-based and culturally appropriate types of health-and-wellness programs/intervention are important in reducing the prevalence, incidence, and progression to T2DM-associated complication in Indigenous communities (Harris et al. [9]). One method an individual can assess the effectiveness of a health-and-wellness program is through self-monitoring of blood glucose. Self-monitoring of blood glucose can be performed through a reflectance-blood-glucose meter, or a flash glucose monitor, or a continuous glucose monitor [33]. The blood-glucose information can be used by the individual to adjust behavior, or self-adjust glycemic medication to improve blood-glucose control [33]. However, health-and-wellness programs potentially impacts more than blood glucose, and the effectiveness of programs should ideally be assessed on a suite of variables (Table 4). Other metabolic risk factors, such as larger waist circumference (or other measure of obesity), elevated triglycerides, reduced HDL cholesterol, and elevated blood pressure, are also important with respect to the development and control of T2DM complications [28,29,30]. However, the results of the present study are suggestive that not all risk factors considered for non-Indigenous populations are appropriate for James Bay Cree, such as diastolic blood pressure and the standard lipid profile suite. Thus, an issue exists when there is a suite of variables being considered [18,19] by a patient and/or researcher with respect to assessing the effectiveness of a health-and-wellness program/intervention. 

The more basic DFA models (1–4) are enhanced self-monitoring of blood glucose protocols for patients, because the variables age, blood glucose, obesity (waist girth or BMI), and blood pressure does not require the assistance of a health clinician after the patient has been initially diagnosed as T2DM by a health-care clinician, and the patient properly trained in variable-measurement protocol. Further, BMI calculators are readily available online (e.g., National Institutes of Health BMI Calculator [34]). Even the variables used in models 5 to 7 can be obtained at point-of-contact—using the less invasive finger stick—without the need for venipuncture and blood samples to be sent to the laboratory. Indeed, point-of-contact systems, such as, the Cholestech LDX system cartridge for lipid profile (total cholesterol, HDL-cholesterol, triglycerides) and fasting glucose determination can be done in a 5 min time duration [35,36]. In addition, the Cholestech LDX point-of contact analytical device determinations have been shown to be comparable in performance to laboratory-derived results [35,36], with proven performance in “real-world” remote Indigenous communities [37]. Point-of-contact testing especially in underserviced populations has several advantages over laboratory testing, such as, on-the-spot test results to expedite decision-making, plus the convenience for people being assessed [33]. Thus, DFA models can be used by researchers in assessing the effectiveness of health-and-wellness programs in rural and remote Indigenous communities with respect to T2DM, beyond the single variable measurement of fasting blood glucose to include T2DM risk factors associated with the development of T2DM complications. 

Adding further, the immediacy of self-monitoring and point-of-care results would be empowering to T2DM patients, because maintaining the status quo in some cases or small improvements in others along the DFE continuum over time may be as important as achieving management targets, since a relationship could be built between participant and researcher [31]. This would especially be true when assessing the effectiveness of on-the-land and other culturally-appropriate programs in Indigenous communities [31]. 

### Limitations

The major limitation of the DFA and diabetes classification tools is that the tool is only valid for the population on which the tool was derived [38,39]. However, the T2DM-classification approaches that have been published can be extended to other populations (e.g., Iranian (Tapak et al. [40], Habibi et al. [41]); Chinese (Chen and Pan, [42]); Indonesian (Rahayu et al. [43])) to generate their own T2DM-classification system [39]. Thus, there is the potential for pan-Indigenous generalizability of the DFA approach, as long as an appropriate dataset is used. An Indigenous-and-people-specific dataset is required to account for the heterogeneity in genetic, cultural, and geographic factors between Indigenous communities within Canada, and around the world. 

It should also be mentioned that for clinical practice, the usefulness of the DFA models would be more limited, especially where HbA1c is used preferentially. HbA1c was not incorporated in any of the DFA models, because of the reported high prevalence of Fe-deficiency anemia in Canadian Indigenous populations [44,45]. To the point, since Fe-deficiency anemia is relatively common in James Bay Cree throughout the life course [46,47]—and Fe-deficiency anemia impacts the accuracy of HbA1c measurement [48,49,50]—the use of HbA1c as a marker raises some concern with its use with this population.

Lastly, culturally-appropriate health-and-wellness programs in First Nations communities have benefits beyond the measured biomedical variables incorporated in the DFA tool—and any comprehensive evaluation of the effectiveness of an intervention should also consider these positive benefits (e.g., mental, emotional, socio-cultural including spiritual)—in conjunction with the biomedical. Thus, evaluation of health-and-wellness programs should ideally include not only a biomedical perspective, but also an Indigenous perspective of the impacts of a program. Each perspective would contribute to a better understanding of a complex issue; that is, the different perspectives would be complementary to each other [24]. 

## 5. Conclusions

Other discriminating tools have been developed—such as, the Diabetes Risk Calculator based on the 1988–1994 US National Health and Nutrition Examination Survey (NHANES) dataset (Heikes et al. [38]) and T2DM-related variables (e.g., height, weight, waist circumference, age, sex, race/ethnicity, gestational diabetes, high blood pressure, high cholesterol, history of diabetes in any blood relative)—while the Diabetes Classifier used the 1999–2004 US NHANES dataset and a similar set of variables (e.g., height, weight, waist circumference, BMI, age, gender, race and ethnicity, hypertension, family history of T2DM, physical activity; Yu et al. [39]). Neither of these T2DM risk-related tools included fasting blood glucose as a variable, by design; the Diabetes Risk Calculator [38] and the Diabetes Classifier [39] were developed as non-invasive T2DM-screening tools. Thus, neither of these tools would be useful in assessing the effectiveness of a health-and-wellness program, because they were not designed to do so. Furthermore, we found, as expected, that blood glucose is the most important discriminating variable in all DFA models (Table 4). The high specificity of all the models (Figure 2) highlights the potential of these DFA models to be used in the assessment of the effectiveness of health-and-wellness programs using a suite of variables to chart overall improvement of an individual along a continuum, in First Nations communities. However, our DFA tool was developed using a James Bay Cree database; thus, it is most appropriate to assess health-and-wellness programs (including T2DM interventions) in the James Bay Cree population. 

## Figures and Tables

**Table 1:** Descriptive statistics for female (*n* = 440) and male (*n* = 315) participants, with and without diagnosed type 2 diabetes mellitus (T2DM).

Sex	Variable						95% C.I. for Mean			
		T2DM	*N*	Median	Geometric Mean	Mean	Lower Bound	Upper Bound	Minimum	Maximum
Female	Age (year)	Yes	111	48.00	46.85	48.97	46.28	51.66	25.00	91.00
		No	329	36.00	36.47	38.72	37.19	40.25	21.00	88.00
	Waist Girth (cm)	Yes	96	119.75	120.67	121.71	118.41	125.01	92.50	172.00
		No	324	111.25	109.90	110.87	109.30	112.45	70.00	147.00
	BMI (kg/m^2^)	Yes	94	38.02	37.90	38.75	37.00	40.51	24.60	69.60
		No	323	34.26	33.90	34.53	33.82	35.24	16.90	58.90
	Systolic BP (mm Hg; mean of 2nd and 3rd)	Yes	96	122.00	122.43	123.73	120.06	127.40	65.00	185.00
		No	325	117.00	117.61	118.41	116.89	119.94	85.00	180.00
	Diastolic BP (mm Hg; mean of 2nd and 3rd)	Yes	96	72.75	71.06	72.13	69.69	74.56	37.00	104.00
		No	324	73.00	71.60	72.41	71.25	73.57	40.00	100.00
	Biochemistry: Glucose (mmol/L)	Yes	98	7.80	8.70	9.35	8.61	10.08	4.00	18.30
		No	326	5.40	5.55	5.68	5.51	5.84	3.00	21.80
	Biochemistry: Triglycerides (mmol/L)	Yes	98	1.81	1.83	2.06	1.82	2.30	0.72	9.35
		No	326	1.26	1.30	1.41	1.34	1.48	0.39	5.65
	Biochemistry: Cholesterol HDL (mmol/L)	Yes	98	1.19	1.17	1.20	1.15	1.26	0.70	1.86
		No	326	1.26	1.27	1.31	1.27	1.35	0.66	3.26
	Biochemistry: Cholesterol (mmol/L)	Yes	98	4.41	4.38	4.47	4.28	4.65	2.50	8.70
		No	326	4.43	4.45	4.53	4.43	4.62	2.30	8.19
Male	Age (year)	Yes	52	58.00	54.31	56.42	52.33	60.51	25.00	89.00
		No	263	39.00	38.72	41.08	39.30	42.86	21.00	87.00
	Waist Girth (cm)	Yes	48	117.00	117.64	118.27	114.65	121.89	83.00	163.00
		No	253	109.00	108.90	109.97	108.07	111.86	40.00	202.00
	BMI (kg/m^2^)	Yes	46	33.83	34.06	34.46	32.82	36.09	22.00	56.40
		No	246	31.28	31.18	31.66	30.96	32.36	20.00	54.70
	Systolic BP (mm Hg; mean of 2nd and 3rd)	Yes	48	125.50	129.03	130.11	125.05	135.18	105.00	175.00
		No	253	122.00	123.49	124.17	122.51	125.84	95.00	196.00
	Diastolic BP (mm Hg; mean of 2nd and 3rd)	Yes	48	75.50	75.48	76.00	73.42	78.58	56.00	97.00
		No	253	78.00	76.57	77.26	76.00	78.52	45.00	112.50
	Biochemistry: Glucose (mmol/L)	Yes	51	8.00	8.77	9.36	8.29	10.43	5.00	24.40
		No	256	5.50	5.63	5.72	5.56	5.87	4.20	16.80
	Biochemistry: Triglycerides (mmol/L)	Yes	50	1.58	1.60	1.76	1.53	1.98	0.45	4.58
		No	256	1.36	1.43	1.70	1.51	1.89	0.45	21.35
	Biochemistry: Cholesterol HDL (mmol/L)	Yes	50	1.16	1.11	1.16	1.08	1.24	0.15	1.81
		No	256	1.16	1.16	1.19	1.16	1.23	0.64	2.36
	Biochemistry: Cholesterol (mmol/L)	Yes	50	4.28	4.15	4.23	3.99	4.46	2.70	6.79
		No	256	4.88	4.85	4.94	4.82	5.05	2.66	7.85

BMI = Body mass index, BP = Blood pressure, C.I. = Confidence interval, HDL = High-density lipoprotein.

**Table 2:** Wilk’s Lambda tests of equality of group (type 2 diabetes mellitus, T2DM and non-T2DM) means: *p*-values for F-test.

Sex	Model Variables	Model 1	Model 2	Model 3	Model 4	Model 5	Model 6 (AHA MetS with Waist)	Model 7 (MetS with BMI)
Female	Age (year)	**<0.0005**	**<0.0005**	**<0.0005**	**<0.0005**	**<0.0005**	~	~
	Glucose [mmol/L]	**<0.0005**	**<0.0005**	**<0.0005**	**<0.0005**	**<0.0005**	**<0.0005**	**<0.0005**
	BMI [kg/m^2^]	~	**<0.0005**	~	**<0.0005**	**<0.0005**	~	**<0.0005**
	Waist Girth [cm]	~	~	**<0.0005**	~	~	**<0.0005**	~
	Mean 2,3 Systolic BP [mm Hg]	~	~	~	**0.008**	**0.008**	**0.005**	**0.008**
	Mean 2,3 Diastolic BP [mm Hg]	~	~	~	0.753	0.753	0.805	0.753
	HDL Cholesterol [mmol/L]	~	~	~	~	**0.004**	**0.003**	**0.004**
	Triglycerides [mmol/L]	~	~	~	~	**<0.0005**	**<0.0005**	**<0.0005**
	Cholesterol [mmol/L]	~	~	~	~	0.523	~	~
	Sample size (*n*)	424	415	418	414	414	417	414
Male	Age (year)	**<0.0005**	**<0.0005**	**<0.0005**	**<0.0005**	**<0.0005**	~	~
	Glucose [mmol/L]	**<0.0005**	**<0.0005**	**<0.0005**	**<0.0005**	**<0.0005**	**<0.0005**	**<0.0005**
	BMI [kg/m^2^]	~	**<0.0005**	~	**<0.0005**	**<0.0005**	~	**0.001**
	Waist Girth [cm]	~	~	**<0.0005**	~	~	**<0.0005**	~
	Mean 2,3 Systolic BP [mm Hg]	~	~	~	**0.007**	**0.003**	**0.006**	**0.003**
	Mean 2,3 Diastolic BP [mm Hg]	~	~	~	0.607	0.756	0.605	0.756
	HDL Cholesterol [mmol/L]	~	~	~	~	0.917	0.591	0.917
	Triglycerides [mmol/L]	~	~	~	~	0.641	0.509	0.641
	Cholesterol [mmol/L]	~	~	~	~	**<0.0005**	~	~
	Sample size (*n*)	307	291	300	291	290	299	290

Note: cell value of ~ indicates variable not used in model. AHA = American Heart Association, BMI = Body mass index, BP = Blood pressure, HDL = High-density lipoprotein, MetS = Metabolic Syndrome.

**Table 3:** Canonical Discriminant Function Coefficients (unstandardized).

Sex	Model Variables	Model 1	Model 2	Model 3	Model 4	Model 5	Model 6 (AHA MetS with Waist)	Model 7 (MetS Variables with BMI)
Female	Age (year)	0.010	0.008	0.008	0.005	0.008	~	~
	Glucose [mmol/L]	10.210	9.828	9.637	9.824	8.708	9.050	9.222
	BMI [kg/m^2^]	~	2.728	~	2.625	2.037	~	2.176
	Waist Girth [cm]	~	~	4.197	~	~	3.396	~
	Mean 2,3 Systolic BP [mm Hg]	~	~	~	2.199	2.022	2.972	2.868
	Mean 2,3 Diastolic BP [mm Hg]	~	~	~	−1.489	−1.464	−1.916	−2.118
	HDL Cholesterol [mmol/L]	~	~	~	~	2.419	0.916	0.815
	Triglycerides [mmol/L]	~	~	~	~	3.613	2.538	2.554
	Cholesterol [mmol/L]	~	~	~	~	−3.398	~	~
	constant	−9.156	−12.959	−17.184	−14.471	−12.159	−18.652	−14.564
	Wilk’s Lambda Test of Function (Chi-square significance)	**<0.0005**	**<0.0005**	**<0.0005**	**<0.0005**	**<0.0005**	**<0.0005**	**<0.0005**
Male	Age (year)	0.023	0.028	0.023	0.026	0.023	~	~
	Glucose [mmol/L]	11.116	11.046	10.795	11.035	10.245	12.042	12.548
	BMI [kg/m^2^]	~	0.665	~	0.706	1.704	~	2.116
	Waist Girth [cm]	~	~	1.513	~	~	3.111	~
	Mean 2,3 Systolic BP [mm Hg]	~	~	~	1.043	0.196	3.581	4.053
	Mean 2,3 Diastolic BP [mm Hg]	~	~	~	−1.245	0.060	−2.557	−2.786
	HDL Cholesterol [mmol/L]	~	~	~	~	5.041	1.769	1.621
	Triglycerides [mmol/L]	~	~	~	~	1.078	−1.136	−1.320
	Cholesterol [mmol/L]	~	~	~	~	−7.302	~	~
	constant	−10.425	−11.519	−13.238	−11.348	−9.294	−19.394	−17.056
	Wilk’s Lambda Test of Function (Chi-square significance)	**<0.0005**	**<0.0005**	**<0.0005**	**<0.0005**	**<0.0005**	**<0.0005**	**<0.0005**

Note: cell value of ~ indicates variable not used in model. AHA = American Heart Association, BMI = Body mass index, BP = Blood pressure, HDL = High-density lipoprotein, MetS = Metabolic Syndrome.

**Table 4:** Canonical discriminant function coefficients (standardized).

Sex	Model Variables	Model 1	Model 2	Model 3	Model 4	Model 5	Model 6 (AHA MetS with Waist)	Model 7 (MetS with BMI)
Female	Age (year)	0.141	0.116	0.110	0.072	0.107	~	~
	Glucose [mmol/L]	0.959	0.923	0.906	0.924	0.819	0.852	0.867
	BMI [kg/m^2^]	~	0.235	~	0.226	0.175	~	0.187
	Waist Girth [cm]	~	~	0.244	~	~	0.198	~
	Mean 2,3 Systolic BP [mm Hg]	~	~	~	0.118	0.108	0.160	0.154
	Mean 2,3 Diastolic BP [mm Hg]	~	~	~	−0.102	−0.100	−0.132	−0.145
	HDL Cholesterol [mmol/L]	~	~	~	~	0.146	0.056	0.049
	Triglycerides [mmol/L]	~	~	~	~	0.403	0.282	0.285
	Cholesterol [mmol/L]	~	~	~	~	−0.232	~	~
Male	Age (year)	0.327	0.380	0.335	0.359	0.314	~	~
	Glucose [mmol/L]	0.891	0.848	0.864	0.847	0.787	0.965	0.964
	BMI [kg/m^2^]	~	0.049	~	0.053	0.127	~	0.158
	Waist Girth [cm]	~	~	0.090	~	~	0.185	~
	Mean 2,3 Systolic BP [mm Hg]	~	~	~	0.049	0.009	0.168	0.191
	Mean 2,3 Diastolic BP [mm Hg]	~	~	~	−0.072	0.003	−0.147	−0.161
	HDL Cholesterol [mmol/L]	~	~	~	~	0.267	0.093	0.086
	Triglycerides [mmol/L]	~	~	~	~	0.153	−0.159	−0.187
	Cholesterol [mmol/L]	~	~	~	~	−0.500	~	~

AHA American Heart Association, BMI = Body mass index, BP = Blood pressure, HDL = High-density lipoprotein, MetS = Metabolic Syndrome.

**Figure A1:**
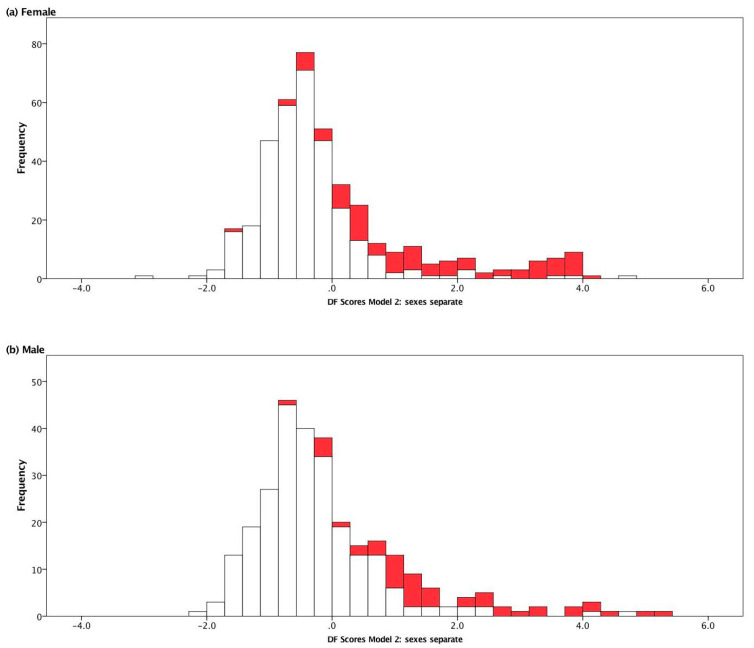
Histogram of scores from Model 2 discriminant functions analysis, for (**a**) females and (**b**) males. Solid bars: Type 2 diabetes mellitus (T2DM) participants; open bars: Non-T2DM participants.

## References

[1] Scully T. (2012). Diabetes in numbers. Nature.

[2] American Diabetes Association (2017). Classification and diagnosis of diabetes. Diabetes Care.

[3] Chen L., Magliano D.J., Zimmet P.Z. (2012). The worldwide epidemiology of type 2 diabetes mellitus-Present and future perspectives. Nat. Rev. Endocrinol..

[4] Yu C.H.Y., Zinman B. (2007). Type 2 diabetes and impaired glucose tolerance in aboriginal populations: A global perspective. Diabetes Res. Clin. Pract..

[5] Gracey M., King M. (2009). Indigenous health part 1: Determinants and disease patterns. Lancet.

[6] Government of Canada (2011). Chapter 6: Diabetes in Canada: Facts and Figures from a Public Health Perspective–First Nations, Inuit, and Métis-Canada.ca.

[7] Institute of Health Economics Diabetes Care and Management in Indigenous Populations in Canada—A Pan-Canadian Policy Roundtable. https://www.ihe.ca.

[8] Leung L. (2016). Diabetes mellitus and the Aboriginal diabetic initiative in Canada: An update review. J. Fam. Med. Prim. Care.

[9] Harris S.B., Bhattacharyya O., Dyck R., Hayward M.N., Toth E.L. (2013). Type 2 Diabetes in Aboriginal Peoples. Can. J. Diabetes.

[10] Halseth R. (2019). The Prevalence of Type 2 Diabetes among First Nations and Considerations for Prevention.

[11] Discriminant Analysis, A Powerful Classification Technique in Data Mining. https://www.lexjansen.com/wuss/2001/WUSS01036.pdf.

[12] Hou S., Riley C.B. (2015). Is uncorrelated linear discriminant analysis really a new method?. Chemom. Intell. Lab. Syst..

[13] Burns R.B., Burns R.A. (2008). Business Research Methods and Statistics Using SPSS.

[14] Lakowski R., Aspinall P.A., Kinnear P.R. (1972). Association between colour vision losses and diabetes mellitus. Ophthalmic Res..

[15] Amoah E., Glickman J.L., Malchoff C.D., Sturgill B.C., Kaiser D.L., Bolton W.K. (1988). Clinical identification of nondiabetic renal disease in diabetic patients with type I and type II disease presenting with renal dysfunction. Am. J. Nephrol..

[16] Rondinelli R.D., Robinson L.R., Hassanein K.M., Stolov W.C., Fujimoto W.Y., Rubner D.E. (1994). Further studies on the electrodiagnosis of diabetic peripheral polyneuropathy using discriminant function analysis. Am. J. Phys. Med. Rehabil..

[17] Kordonouri O., Danne T., Hopfenmüller W., Enders I., Hövener G., Weber B. (1996). Lipid profiles and blood pressure: Are they risk factors for the development of early background retinopathy and incipient nephropathy in children with insulin-dependent diabetes mellitus?. Acta Paediatr. Int. J. Paediatr..

[18] Polat K., Güneş S., Arslan A. (2008). A cascade learning system for classification of diabetes disease: Generalized Discriminant Analysis and Least Square Support Vector Machine. Expert Syst. Appl..

[19] Dogantekin E., Dogantekin A., Avci D., Avci L. (2010). An intelligent diagnosis system for diabetes on Linear Discriminant Analysis and Adaptive Network Based Fuzzy Inference System: LDA-ANFIS. Digit. Signal Process. A Rev. J..

[20] Nai-Arun N., Punnee S. (2014). Ensemble learning model for diabetes classification. Adv. Mater. Res..

[21] Muller P.S., Nirmala M. (2017). Identifying Most Influential Risk Factors of Gestational Diabetes Mellitus Using Discriminant Analysis. Int. J. Pure Appl. Math..

[22] Shirley Muller P., Nirmala M. (2018). Effects of pre-pregnancy maternal body mass index on gestational diabetes mellitus. Int. J. Eng. Technol..

[23] Tran T.S., Hirst J.E., Do M.A.T., Morris J.M., Jeffery H.E. (2013). Early prediction of gestational diabetes mellitus in Vietnam. Diabetes Care.

[24] Tsuji L., Ho E. (2002). Traditional environmental knowledge and western science: In search of common ground. Can. J. Native Stud..

[25] Subedi R., Roshanafshar S., Lawson Greenberg T. (2020). Developing Meaningful Categories for Distinguishing Levels of Remoteness in Canada Analytical Studies: Methods and References.

[26] Liberda E.N., Zuk A.M., Tsuji L.J.S. (2019). Complex contaminant mixtures and their associations with intima-media thickness. BMC Cardiovasc. Disord..

[27] Yi L.Z., Yuan D.L., Che Z.H., Liang Y.Z., Zhou Z.G., Gao H.Y., Wang Y.M. (2008). Plasma fatty acid metabolic profile coupled with uncorrelated linear discriminant analysis to diagnose and biomarker screening of type 2 diabetes and type 2 diabetic coronary heart diseases. Metabolomics.

[28] Rask-Madsen C., Kahn C.R. (2012). Tissue–Specific Insulin Signaling, Metabolic Syndrome, and Cardiovascular Disease. Arterioscler. Thromb. Vasc. Biol..

[29] Yamagishi K., Iso H. (2017). The criteria for metabolic syndrome and the national health screening and education system in Japan. Epidemiol. Health.

[30] Grundy S.M., Brewer H.B., Cleeman J.I., Smith S.C., Lenfant C. (2004). Definition of Metabolic Syndrome: Report of the National Heart, Lung, and Blood Institute/American Heart Association Conference on Scientific Issues Related to Definition. Circulation.

[31] Crowshoe L., Dannenbaum D., Green M., Henderson R., Hayward M.N., Toth E. (2018). Type 2 Diabetes and Indigenous Peoples. Can. J. Diabetes.

[32] Smits N. (2010). A note on Youden’s J and its cost ratio. BMC Med. Res. Methodol..

[33] Berard L.D., Siemens R., Woo V. (2018). Monitoring Glycemic Control-Diabetes Canada Clinical Practice Guidelines Expert Committee. Can. J. Diabetes.

[34] National Institutes of Health Calculate Your BMI-Standard BMI Calculator. https://www.nhlbi.nih.gov/health/educational/lose_wt/BMI/bmicalc.htm.

[35] Whitehead S.J., Ford C., Gama R. (2014). A combined laboratory and field evaluation of the Cholestech LDX and CardioChek PA point-of-care testing lipid and glucose analysers. Ann. Clin. Biochem..

[36] Donato L.J., Deobald G.R., Wockenfus A.M., Hornseth J.M., Saenger A.K., Karon B.S. (2015). Comparison of two point of care devices for capillary lipid screening in fasting and postprandial adults. Clin. Biochem..

[37] Shemesh T., Rowley K.G., Shephard M., Piers L.S., O’Dea K. (2006). Agreement between laboratory results and on-site pathology testing using Bayer DCA2000+ and Cholestech LDX point-of-care methods in remote Australian Aboriginal communities. Clin. Chim. Acta.

[38] Heikes K.E., Eddy D.M., Arondekar B., Schlessinger L. (2008). Diabetes risk calculator: A simple tool for detecting undiagnosed diabetes and pre-diabetes. Diabetes Care.

[39] Yu W., Liu T., Valdez R., Gwinn M., Khoury M.J. (2010). Application of support vector machine modeling for prediction of common diseases: The case of diabetes and pre-diabetes. BMC Med. Inform. Decis. Mak..

[40] Tapak L., Mahjub H., Hamidi O., Poorolajal J. (2013). Real-data comparison of data mining methods in prediction of diabetes in Iran. Healthc. Inform. Res..

[41] Habibi S., Ahmadi M., Alizadeh S. (2015). Type 2 Diabetes Mellitus Screening and Risk Factors Using Decision Tree: Results of Data Mining. Glob. J. Health Sci..

[42] Chen P., Pan C. (2018). Diabetes classification model based on boosting algorithms. BMC Bioinform..

[43] Rahayu W., Santi V.M., Putri B.S. (2019). Classification of diabetes events using discriminant analysis. J. Phys. Conf. Ser..

[44] Christofides A., Schauer C., Zlotkin S.H. (2005). Iron deficiency anemia among children: Addressing a global public health problem within a Canadian context. Paediatr. Child. Health.

[45] Tahir E., Ayotte P., Little M., Bélanger R.E., Lucas M., Mergler D., Laouan Sidi E.A., McHugh N.G.L., Lemire M. (2020). Anemia, iron status, and associated protective and risk factors among children and adolescents aged 3 to 19 years old from four First Nations communities in Quebec. Can. J. Public Health.

[46] Delormier T., Kuhnlein H.V. (1999). Dietary characteristics of Eastern James Bay Cree women. Arctic.

[47] Willows N., Dannenbaum D., Vadeboncoeur S. (2012). Prevalence of anemia among Quebec Cree infants from 2002 to 2007 compared with 1995 to 2000. Can. Fam. Physician.

[48] Coban E., Ozdogan M., Timuragaoglu A. (2004). Effect of iron deficiency anemia on the levels of hemoglobin A1c in nondiabetic patients. Acta Haematol..

[49] Hardikar P.S., Joshi S.M., Bhat D.S., Raut D.A., Katre P.A., Lubree H.G., Jere A., Pandit A.N., Fall C.H.D., Yajnik C.S. (2012). Spuriously high prevalence of prediabetes diagnosed by HbA 1c in young Indians partly explained by hematological factors and iron deficiency anemia. Diabetes Care.

[50] Guo W., Zhou Q., Jia Y., Xu J. (2019). Increased levels of glycated hemoglobin A1c and iron deficiency anemia: A review. Med. Sci. Monit..

